# MBBC: an efficient approach for metagenomic binning based on clustering

**DOI:** 10.1186/s12859-015-0473-8

**Published:** 2015-02-05

**Authors:** Ying Wang, Haiyan Hu, Xiaoman Li

**Affiliations:** 1 Department of Electric Engineering and Computer Science, University of Central Florida, Orlando, FL 32816 USA; 2 Burnett School of Biomedical Science, University of Central Florida, Orlando, FL 32816 USA

**Keywords:** Metagenomics, Binning, Taxonomy-independent, EM Algorithm, Markov properties

## Abstract

**Background:**

Binning environmental shotgun reads is one of the most fundamental tasks in metagenomic studies, in which mixed reads from different species or operational taxonomical units (OTUs) are separated into different groups. While dozens of binning methods are available, there is still room for improvement.

**Results:**

We developed a novel taxonomy-independent approach called MBBC (**M**etagenomic **B**inning **B**ased on **C**lustering) to cluster environmental shotgun reads, by considering k-mer frequency in reads and Markov properties of the inferred OTUs. Tested on twelve simulated datasets, MBBC reliably estimated the species number, the genome size, and the relative abundance of each species, independent of whether there are errors in reads. Tested on multiple experimental datasets, MBBC outperformed two state-of-the-art taxonomy-independent methods, in terms of the accuracy of the estimated species number, genome sizes, and percentages of correctly assigned reads, among other metrics.

**Conclusions:**

We have developed a novel method for binning metagenomic reads based on clustering. This method is demonstrated to reliably predict species numbers, genome sizes, relative species abundances, and k-mer coverage in simple datasets. Our method also has a high accuracy in read binning. The MBBC software is freely available at http://eecs.ucf.edu/~xiaoman/MBBC/MBBC.html.

**Electronic supplementary material:**

The online version of this article (doi:10.1186/s12859-015-0473-8) contains supplementary material, which is available to authorized users.

## Background

Binning environmental shotgun reads is critical to metagenomic studies [1,2]. In a metagenomics project, genome sequences of different species from an environmental sample are randomly cut into short DNA fragments and then sequenced [1-3]. The sequenced DNA fragments are often called reads, and the mixed reads from different species in an environment are thus designated as environmental shotgun reads [2]. Because the information of the species origin of reads and the relative order of reads in the genomes is lost during sequencing, it is crucial to cluster the mixed environmental shotgun reads into reads from the same species or operational taxonomical units (OTUs), so called “binning reads” [2]. By binning reads, researchers can identify the number and the abundances of species in the environment, and further understand what functional roles each species plays and how these species work together, which are critical for the study of microbes.

Many computational methods have been developed to bin environmental shotgun reads [4-22]. These methods can be broadly classified into two categories. One category is taxonomy-dependent [5,8-10,16-21,23,24], in which one queries reads in reference databases and utilizes the origin of the hit sequences in reference databases to bin reads. The reference databases commonly used include the non-redundant nucleotide database at the National Center for Biotechnology Information (NCBI), Uniprot [25], Pfam [26], etc. The other category of methods is taxonomy-independent [4,7,11-13,27,28], in which the composition information of reads is used to group reads. The rationale behind taxonomy-independent methods is that reads from different species have different composition properties. For instance, different *α* -proteobacteria species have GC contents ranging from <30% to >60% [29]. In addition to GC content, the frequency of tetranucleotides and other features in reads are also commonly used as the composition information of reads [11,22,30].

Despite the existence of many read-binning methods, there is much room for improvement [22]. The taxonomy-dependent methods are hampered by the limited number of sequenced microbial genomes, more than 99% of which are still unknown and unstudied [31]. The taxonomy-independent methods also have various problems. Early taxonomy-independent methods cannot bin short reads from next generation sequencing technologies [6,31]. Recently, a few methods [6,14,15] have been developed to bin reads, including short reads. For instance, AbundanceBin [14] utilizes the property that k-mers (k base pair long DNA segments) in reads from the same genome have similar frequencies to group reads. Although these methods have been shown to perform well in certain simulated and experimental datasets, recent studies indicate their limitations [22]. One such limitation is that multiple reads have seldom been considered simultaneously to infer their properties other than k-mer frequency. We infer that properties shared by a group of reads are likely useful to cluster short environmental shotgun reads, as demonstrated in the following analyses.

We developed a novel approach called Metagenomic Binning Based on Clustering (MBBC). MBBC first groups reads based on k-mer frequencies within the reads by an expectation maximization (EM) algorithm [32]. The rationale behind this step is that species with different genome coverage usually have different k-mer frequencies and k-mers in reads from the same species often occur similar number of times. Therefore, k-mer frequencies in reads help to separate reads from different species. From the initially grouped reads, MBBC then infers the Markov properties of reads within each group, under the assumption that the majority of reads with similar k-mer frequencies are likely from the same genome and therefore from the same Markov chain. Finally, MBBC iteratively clusters reads based on the learned Markov properties and infers the Markov properties of reads in the same groups until the process converges. Tested on twelve simulated datasets, MBBC reliably clustered reads and determined the species number, genome sizes, and k-mer coverage of each species. The k-mer coverage of a species in this study is the average number of reads covering a random k-mer in the genome of this species, which is an approximation of the genome coverage that is calculated as the sum of the length of all reads from this species divided by the genome length of this species. Tested on multiple real experimental datasets, four of which used 75 base pair long short reads, MBBC performed the same or better than two state-of-the-art taxonomy-independent methods [14,15]. MBBC is thus a useful method for metagenomic studies.

## Results

### MBBC reliably estimates the species number, genome sizes, relative species abundances, and k-mer coverage

We applied MBBC to twelve simulated datasets with the initial species number, *m*, set at 10. These datasets used species from three randomly selected genera, from each of which four species were randomly selected (Additional file 1). We observed that in each dataset, MBBC predicted the exact species number (Additional file 2). In all datasets, regardless of whether the genome coverage ratio was larger or smaller than 2 and whether there were errors in reads, the predicted genome size, relative species abundance, and k-mer coverage were close to the actual ones (Additional file 2).

**Figure 1:**
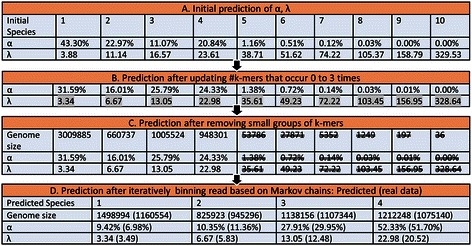
**An example of binning reads from four species in the genus of** ***Spiroplasma*** **by MBBC.** *α* and *λ* represents the estimated relative species abundance and k-mer coverage, respectively. The real genome sizes, *α* and *λ* are listed in the parentheses of the last table in the figure. After updating k-mer occurrences for k-mers occurring fewer than 4 times, the estimated *α* becomes more accurate. After removing small groups, the estimated species number and *α* become more accurate.

Figure 1 provided a detailed example of binning reads from four species in the genus *Spiroplasma* by MBBC (Figure 1). In this example, the genome coverage of the four species was 4, 8, 18, and 32, respectively. MBBC correctly determined the species number. It also reliably predicted the k-mer coverage as 3.34, 6.67, 13.05, and 22.98, respectively, which were close to the actual ones (numbers in the parentheses in Figure 1). The actual k-mer coverage was calculated by counting the number of times k-mers in a genome covered by reads from this genome. Moreover, MBBC reasonably estimated the genome sizes for the four species (Figure 1).

It is also evident that two steps in the EM algorithm of the MBBC are important for its accuracy (Figure 1). One step is to estimate the number of k-mers occurring 0, 1, 2, and 3 times in reads. This step is necessary as the observed numbers of k-mers occurring 0 to 3 times are likely biased due to the existence of low abundance species and sequence errors [14,22,33]. In fact, after estimating these numbers by iteratively running the EM algorithm, the estimated k-mer coverage becomes much closer to the actual ones. For instance, the predicted k-mer coverage, λ, for the first four groups was changed from (3.88, 11.14, 16.57, 23.61) to (3.34, 6.67, 13.05, 22.98), respectively, and the actual λ was (3.49, 5.83, 12.48, 20.52). The other step is to remove the small groups of k-mers (the estimated genome sizes corresponding to these groups are smaller than 400,000). By removing these small groups and reassigning k-mers, the estimated species abundance, α, becomes much closer to the actual α. For instance, after this step, α for the first four groups was changed from (31.59%, 16.01%, 25.79%, 24.33%) to (16.93%, 11.55%, 23.09%, 48.43%), respectively, while the actual α was (6.98%, 11.36%, 29.95%, 51.70%). These two steps make the EM algorithm in MBBC different from the one implemented in AbundanceBin [14], which always separates k-mers into two groups, even when reads are from more than two species, and neglects the inaccuracy of the observed numbers of k-mers occurring 0, 1, 2, 3 times in reads.

Figure 1 illustrates the importance of the inferred Markov properties to the accuracy of MBBC as well. It is well known that different microbial genomes often follow different Markov properties [34,35]. Previous studies, such as [11], have utilized these properties to assign reads longer than 1000 base pairs in metagenomic studies. Regarding short reads, such as 75 base pairs long reads, it is unlikely to reasonably infer the Markov properties they may have from individual reads. By assuming that most reads grouped by the EM algorithm are likely from one OTU, we have reliably inferred the Markov properties that most reads in a group follow and further filtered reads from other OTUs. To our knowledge, such a strategy has not been explored before. From Figure 1, it is clear that this strategy significantly improves the accuracy of read clustering, which is shown in the generally more accurate estimation of genome sizes and relative species abundance.

To investigate how the change in genome coverage ratios affects the accuracy of the estimation, we applied MBBC to simulated datasets with all genome coverage ratios larger or smaller than 2, using the first three species in the above example. The above example demonstrated that MBBC reliably estimates the species number, genome sizes, relative species abundance, and k-mer coverage. We noticed that the species number was still accurately predicted even when the genome coverage ratios were smaller than 2 (Table 1). Moreover, as expected, we observed that when the genome coverage ratios were larger than 2, the predicted genome sizes and k-mer coverage were in general closer to the actual ones than those with genome coverage ratios smaller than 2 (Table 1). In addition, the prediction still agreed well when the genome coverage ratios were smaller than 2. For instance, for the third species (*sps*), the predicted genome size, relative species abundance, and k-mer coverage was 1,139,322 base pairs, 0.5202, and 10.95, respectively, whereas the actual one was 1,107,344 base pairs, 0.5541, and 10.53, respectively (Table 1).

**Table 1:** **Prediction by MBBC on datasets with different genome coverage ratios or species composition**

**Datasets**	**Predicted genome sizes**	**Actual genome sizes**	**Predicted relative abundance**	**Actual relative abundance**	**Predicted k-mer coverage**	**Actual k-mer coverage**
spa4spd8sps18spt32	1498994	1160554	9.42%	6.98%	3.34	3.49
	825923	945296	10.35%	11.36%	6.67	5.83
	1138156	1107344	27.91%	29.95%	13.05	12.48
	1212248	1075140	52.33%	51.70%	22.98	20.52
spa4spd8sps18	1281577	1160554	16.16%	14.45%	3.24	3.49
	921307	945296	22.61%	23.53%	6.31	5.83
	1226752	1107344	61.23%	62.02%	12.83	12.48
spa5spd8sps15	1607360	1160554	27.03%	19.36%	4.03	4.01
	682864	945296	20.95%	25.23%	7.36	5.83
	1139322	1107344	52.02%	55.41%	10.95	10.53
spa5baa8sps15	1463372	1160554	21.50%	16.49%	4.13	4.01
	1318685	1596490	30.49%	36.30%	6.51	5.87
	1250815	1107344	48.01%	47.21%	10.80	10.53

Each species in each dataset is named by the first two letters of their genus name, followed by the first letter from the species name and then the genome coverage. The first dataset is the one used in Figure 1.

We also investigated the performance of MBBC with species from different genera. Intuitively, it should be easier to bin reads from species of different genera than those from the same genus, because the Markov properties of genomes from different genera may be more different than those from the same genus. When we replaced the second species in the third example above with a species from another genus, we noticed an improvement in the accuracy of MBBC (Table 1). For instance, the estimated k-mer coverage of the replaced species was 6.51, compared with 5.87, the actual k-mer coverage of this species. Conversely, the estimated k-mer coverage of the second species before replacement was 7.36, compared with the actual k-mer coverage of 5.83. Because two species were from the same genus in this example, we further generated three additional datasets using species from different phyla. We found that the overall accuracy predicted was improved when species were from different phyla instead of the same genus (Additional file 3).

### MBBC reliably assigns reads

In addition to estimating species number, genome sizes, and k-mer coverage, another important task in metagenomic analyses is to group reads from the same OTUs together. We investigated how well MBBC binned reads in twelve simulated datasets, listed in the Additional file 1. We observed that 75% to 91% of reads were correctly binned together, even when there was 1% errors in reads and some genome coverage ratios were smaller than 2 (Additional file 2). The accuracy of the binned reads was calculated by assuming the species to be the group with the majority of its reads and then counting how many reads were correctly assigned to these species. We also noticed that the accuracy was genus dependent, in that the accuracy of the binned reads for simulated datasets from one genus was always higher than that from another genus, regardless of whether the genome coverage ratios were smaller than 2 or there were errors in reads, implying that Markov properties of species in certain genera differed more than those of species in other genera. In addition, the genome coverage ratios affected the accuracy of read binning, in that the accuracy for datasets from the same genus was always lowest when the ratios were smaller than 2.

To further investigate how the genome coverage ratios affected accuracy, we applied MBBC to datasets with different genome coverage ratios. We used the same datasets listed in Table 1. As expected, we observed that the accuracy of read binning decreased when the genome coverage ratios decreased (Additional file 2). We also noticed that the accuracy was improved with species from different genera, although the genome coverage ratios were still smaller than 2, because of the consideration of Markov properties of genomes of different species. For instance, in the last two simulated datasets in Table 1, the accuracy of read binning by MBBC was 85.39%, compared with 82.01%, when species from different genera compared with species from the same genus were used (Additional files 2 and 3).

### MBBC works well in real datasets

We applied MBBC to two simplified real datasets. It is a common practice to use simplified real datasets to test developed binning methods [14,15], because most species present and their abundance in original raw read datasets are unknown. One simplified real dataset was the AMD dataset [3], in which long Sanger reads were used. MBBC correctly predicted the species number as 2. MBBC also almost perfectly predicted the relative abundances of the two species as 29.1% and 70.9%, as compared with the actual relative abundance as 29.03% and 70.97%. Moreover, the predicted k-mer coverage of the two species was 4.03 and 8.16, respectively, which were close to the actual coverage (5.14 and 7.35, respectively). Overall, the accuracy of read binning by MBBC in this dataset was 94.27%.

The other simplified real dataset we applied MBBC to was a human gut dataset composed of 4,684,098 short Illumina raw reads from three microbial species. Unexpectedly, MBBC predicted 4 species (Table 2). We noticed that the majority of reads in both the third and fourth groups were from the same species, the third species. Moreover, the sum of the relative abundance of the third and fourth groups was 72.48%, which was close to the relative abundance of the third species, 69.21%. The other two predicted groups agreed well with the corresponding two real species. For instance, the predicted genome size, relative coverage, and k-mer coverage of the second species were 231555 base pairs, 16.87%, and 10.24, respectively, which concurred well with the actual corresponding numbers, 2249085, 16.67%, and 10.24 (Table 2). The accuracy of read binning by MBBC was 74.80% in this dataset, demonstrating that MBBC works well in datasets with long or short reads.

**Table 2:** **Prediction on the human gut dataset by MBBC**

	**MBBC predictions**				**Actual numbers**		
genome size	3524796	2315047	1745685	2274392	NA	2249085	NA
relative abundance	11.25%	16.87%	23.33%	48.55%	14.12%	16.67%	69.21%
k-mer coverage	4.48	10.24	18.78	30	8.28	10.49	18.49

To understand why MBBC did not automatically combine the third and fourth groups into one predicted species, we examined the mapped reads to the genome corresponding to the third species. We noticed that this genome was almost evenly divided into two halves, with coverage of approximately 18 and 30 for the two halves, respectively. Because both halves were longer than the genome size cutoff (400,000), MBBC considered them as two separate genomes. Since the two groups were from the same genome, we also compared the two Markov models learned from reads from the two halves of the genome. We used the relative entropy to measure the difference of the transition matrix of the two Markov chains. We observed that the relative entropy of the two Markov models was 1.14, which was larger than that of the Markov models of the first two species, which had a relative entropy of 0.68. Thus it makes sense that MBBC considered them to be two separate species. This result also implies that different compositions in different genome regions may contribute to different coverage of these regions in genome sequencing.

### MBBC performs better than AbundanceBin and MetaCluster

We compared MBBC with two widely used taxonomy-independent methods, AbundanceBin [14] and MetaCluster 5.0 [15], in the twelve simulated datasets (Additional file 1) and two simplified real datasets mentioned above. Because AbundanceBin was developed for single-end reads, when paired-end reads were used, we ran AbundanceBin by treating the two paired-end reads as independent reads. Because MetaCluster runs on paired-end read data, we did not apply it to the AMD dataset that used single-end reads [3]. Overall, MBBC outperformed the two methods in terms of the estimated species number, genome sizes, relative species abundance, k-mer coverage, and binning accuracy (Additional file 4).

First, we compared the predicted species number in these fourteen datasets. MBBC predicted the right species number in all except one dataset. AbundanceBin and MetaCluster often cannot predict the right species number (Additional file 4). In the twelve simulated datasets, AbundanceBin and MetaCluster correctly predicted the species number in two and zero datasets, respectively. For the AMD dataset, AbundanceBin predicted the correct number of species. For the gut dataset, MetaCluster predicted 512 groups whereas AbundanceBin failed with only one species output. Because the species numbers were not correctly predicted, it was difficult for the two programs to predict other properties of the datasets, such as the genome sizes, the relative abundance, and the k-mer coverage of each species.

Next, we compared the accuracy of read binning in the fourteen datasets (Table 3). Because AbundanceBin and MetaCluster cannot automatically predict the right species number, we specified the known species number as input for the two programs to output the binned reads. In eleven of the twelve simulated datasets, the accuracy of MBBC was better that of the other two methods, with a median of 15% higher accuracy (Table 3). In the only simulated dataset that MBBC did not achieve the highest accuracy, MBBC had an accuracy of 89%, slightly less than the best accuracy of 90%. AbundanceBin performed better than MetaCluster in all simulated datasets without read errors while MetaCluster performed better than AbundanceBin in simulated datasets with read errors (Table 3). MBBC performed better in terms of estimating genome sizes, relative species abundance, etc. In the two real datasets, we also observed that MBBC had a higher accuracy than the other two methods. For instance, the accuracy of MBBC in the gut dataset was 74.80%, compared with 52.63% and 71.65% by AbundanceBin and MetaCluster, respectively.

**Table 3:** **Binning accuracy of MBBC, AbundanceBin and MetaCluster**

**Datasets**	**MBBC**	**MetaCluster**	**AbundanceBin**
lag5lar11las24	91.34%	82.93%	64.60%
lag4lar7las12	78.97%	77.66%	39.09%
laa4lag8lar15las30	86.43%	83.49%	50.98%
laa4lag8lar15las30 (no errors)	87.13%	85.64%	86.41%
spa4spd9sps18	89.58%	78.68%	63.73%
spa5spd8sps15	82.01%	73.71%	52.44%
spa4spd8sps18spt32	87.35%	72.64%	54.60%
spa4spd8sps18spt32 (no errors)	89.09%	74.43%	90.44%
baa3bab7bac15	79.55%	64.83%	61.11%
baa6bab10bac18	75.80%	45.12%	51.13%
baa5bab10bac18bah30	75.71%	34.48%	39.25%
baa5bab10bac18bah30 (no errors)	79.90%	45.82%	66.25%
human gut dataset	74.94%	71.65%	52.63%
AMD dataset	94.14%	NA	73.42%

Finally, we compared the speed of the three methods to bin reads in the fourteen datasets (Additional file 5). All comparisons were performed on the same computer with the following configuration: Intel® Core™ i5-3210 M CPU @ 2.50GHz and 8G RAM. The stacked bars in the additional file 5 displayed the running time of each method on these datasets. We observed that when species number was unknown, the other two methods usually required much more time. When species number was known, MetaCluster was faster (~36.40%) than MBBC, but it only binned reads, and did not predict parameters such as genome sizes, relative species abundance, etc. The most time-consuming part of MBBC was the step to update the number of k-mers occurring 0, 1, 2, and 3 times in reads. AbundanceBin was slow even when the species number was known. This update process required more time to converge, which occupied nearly half of the total running time. Given that MBBC can predict more parameters than MetaCluster, runs faster than AbundanceBin, and can automatically and accurately predict the species number, MBBC is a useful tool for metagenomic data analyses.

## Discussion

We developed a novel approach called MBBC to bin reads from metagenomics projects. MBBC bins reads by employing two types of read composition properties that have never been considered together previously. Tested on simulated and experimental datasets, we demonstrated that MBBC could reliably determine the species number, genome sizes, relative species abundance, and k-mer coverage. Moreover, MBBC grouped reads from the same species with high accuracy. Compared with two popular taxonomy-independent methods, MBBC performed better in almost every dataset tested, with higher accuracy of read binning in both simulated and real datasets.

The inferred Markov property from the binned reads contributes significantly to the success of MBBC. We demonstrated that the Markov property helped to group reads by exploring the differences among species and genera in the above. The Markov properties also help MBBC work better with errors in reads. This is because the majority positions in a read from a species still follow the Markov properties, despite the existence of a few positions with errors.

The comparison of MBBC with AbundanceBin and MetaCluster may be biased by the parameters we used. Except for specifying *m* as the known species numbers, we used the default values of the other parameters in running AbundanceBin and MetaCluster. It is thus possible that the two tools may produce better results with other parameter choices. However, we believe that MBBC should at least behave similarly to or better than the two methods, as the Markov properties of the grouped reads that are important for correctly binning have not been utilized by the two tools.

We suggest users use a large *m* as the initial species number. However, how should one determine this large initial *m*? An economical approach is to start with *m* as a smaller number such as 10. If no small group is discovered by the EM algorithm, one can increase *m* slightly, such as *m* = 15, until the EM algorithm produces small groups. This process will result in robust binning of reads.

The above analyses were mainly based on the twelve simulated datasets and two simplified real datasets. To demonstrate how well MBBC and others perform on original raw read datasets, we also tested them on the original AMD raw read dataset and an original human gut raw read dataset (Additional file 3). In the original AMD dataset, at least five main species were known to present, which could be grouped into three groups with different abundance. MBBC predicted three OTUs, with reads from three species of similar abundance grouped into the same OTUs. The overall read binning accuracy was 68%, while AbundanceBin did not produce any prediction for this dataset. In the original human gut dataset composed of at least 10 known species, MBBC predicted 5 species. By further studying reads in the predicted species, we found that the predicted 5 species represented two groups of species with similar abundance. The overall read binning accuracy in this dataset was 73.51%. AbundanceBin output “nan” for this dataset, which meant that it could not work on complex datasets. MetaCluster considered most reads as “orphan” and only clustered 28% of reads for this dataset. MBBC thus performed better than AbundanceBin and MetaCluster in these two original real datasets. In addition to the original raw read datasets, we also tested MBBC on the Human Microbiome Project (HMP) real datasets and mock datasets. In the three real datasets and two mock datasets we tested, MBBC did better than AbundanceBin and MetaCluster. However, MBBC failed to distinguish species with similar abundance in a few datasets again. The analysis details of these datasets including the original AMD and gut datasets are in the additional file 3. These analyses demonstrated that although MBBC performed well on datasets with large abundance ratios (around 1.5 or larger) and/or datasets composed of species with distinct Markov properties, the actual metagenomic datasets were much more complicated, with many species of similar abundance (abundance ratios close to 1) and many species with complicated Markov properties (different regions of the same genome have different Markov properties). There is still much room for further methodology improvement.

Two aspects may be considered to further improve MBBC. One is the assumption of the Poisson distribution of the frequency of k-mers in reads. The k-mers in reads from one species may not follow a Poisson distribution exactly, and more suitable distributions may be explored. The other aspect is the assumption of the homogeneity of a microbial genome. We demonstrated that the third species in the simplified gut dataset is not homogeneous, which is why MBBC considered it to be two different species. In the future, a better model will be necessary to take the homogeneity of microbial genomes into account when designing methods.

## Conclusions

We developed a novel method for binning metagenomic reads based on clustering. This method was demonstrated to reliably predict species numbers, genome sizes, relative species abundance, and k-mer coverage in simple datasets. It also displayed a high accuracy in read binning. The free tool implementing the developed method is available at http://eecs.ucf.edu/~xiaoman/MBBC/MBBC.html.

## Methods

### Experimental datasets retrieved

We used two simplified real experimental datasets, three HMP real datasets, two HMP mock datasets, and two original raw read datasets to evaluate the MBBC method. The details of the two simplified real datasets were in the following. The HMP datasets and the original real datasets were described in the additional file 3.

One simplified real dataset was the Acid Mine Drainage (AMD) dataset [3] downloaded from http://www.ncbi.nlm.nih.gov/books/NBK6860/. This dataset contained 180,713 single-end reads, with an average read length of 1005 base pairs long. Following a previous study [6], we used the Figaro software [36] to remove the vector sequences in these reads. For the remaining portions of each read, only the longest contiguous bases whose quality values were > =17 were kept [6]. This filtering resulted in 166,715 reads. These 166,715 reads were then mapped to two dominant species using the MuMmer software [37] with the default parameters. In total, 40499 reads were mapped to the two species and used to test the binning methods.

The other simplified real dataset was the human gut dataset from 15 randomly selected samples (Additional file 1) and downloaded from ftp://public.genomics.org.cn/BGI/gutmeta/High_quality_reads/. There were 257,158,754 paired-end reads in this dataset, each of which was 75 base pairs long. These reads were mapped to the following three species using the software SOAP 2.21 [38]: *Bacteroides uniformis*, *Alistipes putredinis*, and *Ruminococcusbromii L2-63*. These species were used because they were the most abundant species and/or had more complete genome sequences in the gut dataset. The command used to map reads was ./soap –a < reads_a > −b < reads_b > −D < index.files > −o < PE_output > −2 < SE_output>, which allowed two mismatches and indels during mapping. There were 4,684,098 reads mapped to the three genomes and used to test the metagenomic binning methods.

### Simulated datasets generated

To generate simulated datasets, we randomly selected three genera that had more than 20 sequenced species in the NCBI Microbial Genome Database (http://www.ncbi.nlm.nih.gov/genomes/MICROBES/microbial_taxtree.html). The three genera selected were *Lactobacillus*, *Spiroplasma*, and *Bartonella*. Next, from each genus, we randomly selected four species to generate simulated datasets (Additional file 1). Note that it is much more challenging to bin reads from species of the same genus than those from different genera. We then generated paired-end reads using MetaSim [39] for each of the three or four species in a dataset, with the given genome coverage. We specified the read length to be 75 base pairs and simulated the reads with no error or with the empirical error model in MetaSim (~1% error rate). Similarly, we generated three simulated datasets with species from different phyla (Additional file 3).

### The framework of the MBBC method

**Figure 2:**
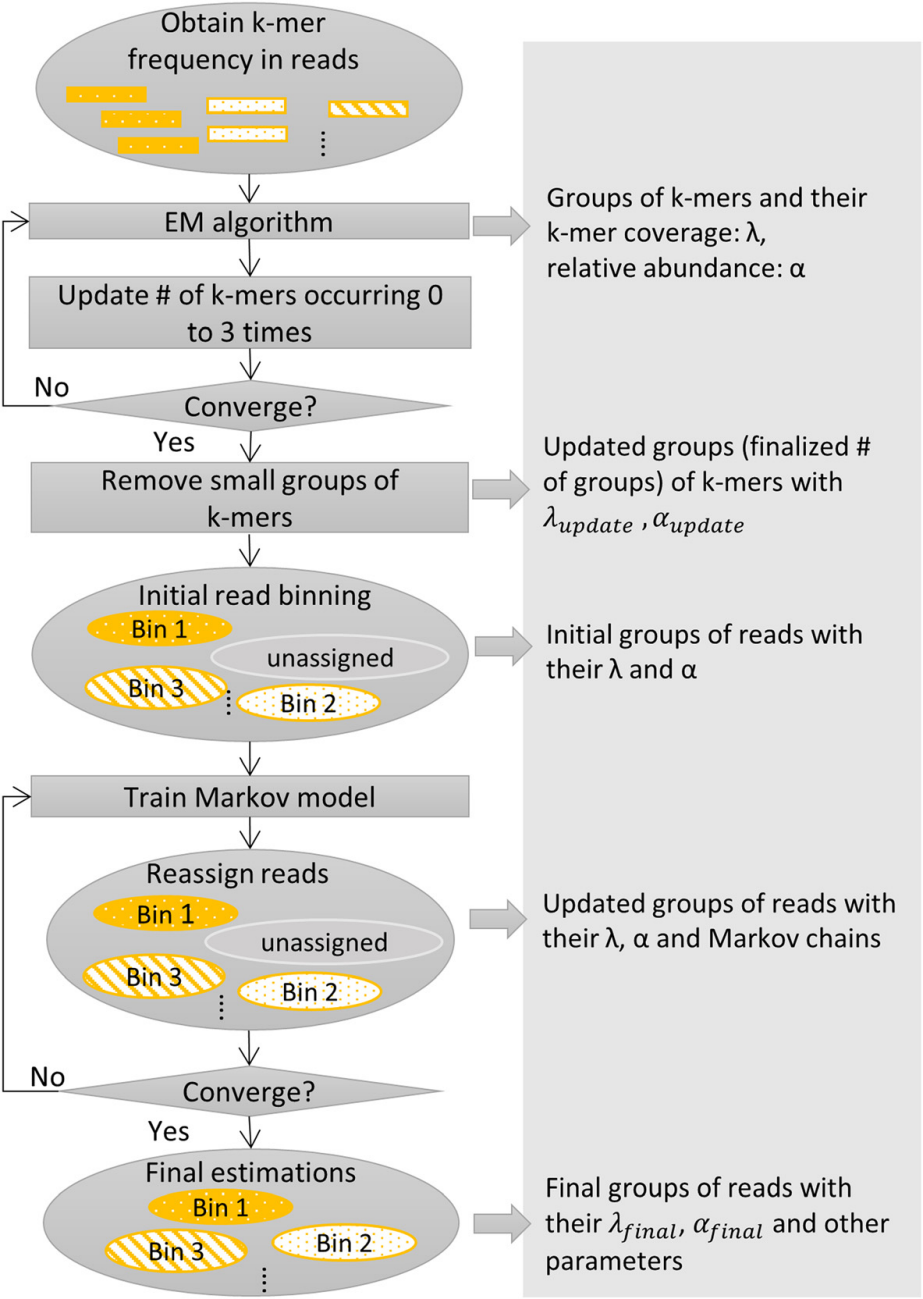
**The procedure of read clustering in MBBC.** The output on the right from each of the main steps on the left is connected with the corresponding steps.

We developed a novel method called MBBC (Figure 2). Our method starts from an EM algorithm to group k-mers in reads based on their frequencies in reads. The assumption behind the k-mer grouping is that the frequency of k-mers in reads follows a mixture of Poisson distributions [33]. Next, MBBC iteratively estimates the number of k-mers that occur 0 to 3 times in reads and runs the EM algorithm to estimate the parameters of the mixed Poisson distributions. The rationale behind the iterative estimation is that these numbers are either unobserved or inaccurate and thus affect the estimation of other parameters [14,15]. Next, MBBC determines the species number and initially groups reads based on the Poisson parameters. MBBC then iteratively models the Markov property of the reads in each group and reassigns reads to groups. Finally, MBBC determines the genome sizes and other metrics based on the assigned reads and the estimated parameters. The details are presented in the following.

### EM algorithm for initial binning of reads

We developed an EM algorithm to group reads based on the frequency of k-mers in reads, where k = 16 is chosen so that the chance that a random k-mer occurs multiple times in a microbial genome is small (<1e-5). The underlying assumption of this EM algorithm is that the frequency of k-mers in reads from a microbial species follows a common distribution. Similar to previous studies [14,33], we use Poisson as the common distribution. Under this assumption, all k-mers in reads from a metagenomic project form the samples of a mixture of Poisson distributions, where the number and the parameters of the Poisson distributions are unknown. EM algorithms are widely used to address mixture problems [33,40], and therefore applied to initially group reads from different Poisson distributions.

The EM algorithm in MBBC assumes that there are in total *n* different k-mers in reads in a metagenomic project that are from *m* different species, where *m* is unknown. Assume that the frequency of these k-mers in all reads, *x*_1_, *x*_2_, …, *x*_*n*_, follows a mixture of *m* Poisson distributions with the unknown parameters *λ*_1_, *λ*_2_, …, *λ*_*m*_. For any i from 1 to *n*, if *x*_*i*_ is from the j-th Poisson distribution, then $P\left({x}_i=x\right)={\alpha}_j{p}_{\mathrm{j}}\left({\lambda}_{\mathrm{j}},x\right)={\alpha}_j\frac{\lambda_j^x}{x!}{e}^{-{\lambda}_j}$, where *α*_*j*_ is the unknown probability that a random k-mer is from the j-th distribution and ${\displaystyle \sum_{j=1}^m{\alpha}_j=1}$. Intuitively, *α*_1_, *α*_2_, …, *α*_*m*_ represent the relative species abundance in the environment, and *λ*_1_, *λ*_2_, …, *λ*_*m*_ represent the k-mer coverage of the species. Because we do not know which distribution *x*_*i*_ is from, we define the missing variable *y*_*i*_, where *y*_*i*_ = *j* indicates that *x*_*i*_ is from the j-th Poisson distribution. With the above notations, the log complete likelihood function of the observed data *X* = {*x*_1_, *x*_2_, …, *x*_*n*_} and the missing data *Y* = {*y*_1_, *y*_2_, …, *y*_*n*_} is $\log \left(L\left(\theta; X,Y\right)\right)={\displaystyle \sum_{i=1}^n \log}\left({\alpha}_{y_i}\ast {p}_{y_i}\left({\lambda}_{y_i},{x}_i\right)\right)$, where the parameter *θ* = {*α*_1_, *α*_2_ ⋯, *α*_*m*_; *λ*_1_, *λ*_2_, ⋯, *λ*_*m*_}. The E-step of the EM algorithm is to calculate *Z*_ij_, which is ${Z}_{\mathrm{ij}}=P\left({y}_i=\mathrm{j}\Big|X,\theta \right)=\frac{\alpha_j\ast {p}_{\mathrm{j}}\left({\lambda}_{\mathrm{j}},{x}_i\right)}{{\displaystyle \sum_{r=1}^m{\alpha}_r\ast {p}_{\mathrm{r}}\left({\lambda}_{\mathrm{r},}{x}_i\right)}}$. The M-step is to estimate the parameters in the following manner: ${\alpha}_j=\frac{1}{n}{\displaystyle \sum_{i=1}^n{Z}_{ij}}$, ${\lambda}_j=\frac{{\displaystyle \sum_{i=1}^n{Z}_{ij}{x}_i}}{{\displaystyle \sum_{i=1}^n{Z}_{ij}}}$.

For a given m, to apply the above EM algorithm, we initialize *α*_*j*_ = 1/*m*, *λ*_*j*_ = *j* * 10 + 10 for j from 1 to m. We then iterate the E-steps and M-steps until the difference between the updated *θ* and the current *θ* is small (<1e-5). Finally, we output the current *θ* = {*α*_1_, *α*_2_ ⋯, *α*_*m*_; ⋅ *λ*_1_, *λ*_2_, ⋯, *λ*_*m*_}and assign k-mers to m different groups based on *θ*.

### Estimation of the species number

The species number m is unknown and required by the above EM algorithm. To estimate *m*, MBBC initializes *m* as a large number so that the output groups from the EM algorithm contain at least a small group that is too small to serve as a k-mer group from a microbial species. To determine whether an output group is small, MBBC first estimates the number of k-mers that occur x = 0, 1, 2, and 3 times, respectively, with the following formula: ${\displaystyle \sum_{j=1}^m\frac{p_{\mathrm{j}}\left({\lambda}_{\mathrm{j}},x\right){\displaystyle \sum_{i=1\&{x}_i\ge 4}^n{Z}_{\mathrm{ij}}}}{1-{\displaystyle \sum_{s=0}^3{p}_{\mathrm{j}}\left({\lambda}_{\mathrm{j}},s\right)}}}$. With the estimated number of k-mers that occur x = 0, 1, 2, and 3 times, MBBC iteratively runs the EM algorithm using the estimated *x*_*i*_ for i from 0 to 3 and the original *x*_*i*_ for i > 3 until the estimated *x*_*i*_ for i < 4 do not change. The rationale to iteratively estimate *x*_*i*_ for i < 4 is that these *x*_*i*_ are inaccurate because of the existence of low abundance species and sequencing errors [14,22,33]. Next, MBBC estimates the genome size represented by each group of k-mers output from the EM algorithm as $\frac{{\displaystyle \sum_{i=1}^{n\hbox{'}}{Z}_{\mathrm{ij}}\ast {x}_i}}{\lambda_{\mathrm{j}}}$, for j from 1 to m, where n’ is used to denote that the estimated k-mers that occur fewer than 4 times are used together with other observed k-mers. Finally, MBBC labels groups of k-mers as small groups if their estimated genome sizes are smaller than 400,000, a cutoff that is smaller than the size of the sequenced smallest genome of living organisms [14], and labels other groups as large groups. With the labelled groups, MBBC estimates the species number as the number of the large groups. The *α*_*j*_ for the large groups is normalized so that their sum is equal to 1. To take the k-mers initially assigned to small groups into account, MBBC then implements one more E-step to calculate *Z*_ij_ and then updates ${\alpha}_j=\frac{1}{n}{\displaystyle \sum_{i=1}^n{Z}_{ij}}$, for i from 1 to n and j from 1 to m.

### Initial read assignment based on the inferred *θ*

With the inferred *θ*, MBBC measures the probability that a read belongs to the j-th species as *p*_j_(*λ*_j_, *x*), for j from 1 to m, where x is the median frequency of the k-mers in this read. MBBC then sorts these probabilities from largest to smallest for each read. For a read, if its largest probability minus the second largest probability is larger than a cutoff C (C = 0.5), this read will be assigned to the species corresponding to the largest probability. When paired-end reads are used in a project, MBBC assigns two paired reads to the same species when at least one read can be assigned and there is no conflict between the assignments of the two reads. In this way, MBBC obtains m + 1 groups of read, one of which corresponds to the unassigned reads. In case that there are more than 50% of reads unassigned, MBBC reduces C by 0.01 and repeats this process until at least half of the reads in the datasets are assigned to the m groups that correspond to m species.

### Final read assignment based on the Markov property

The final assignment of reads is performed by iteratively inferring a 5-th order Markov chain for each group, except for the group corresponding to unassigned reads, and reassigning reads to each group. The rationale of modelling a group of reads by a Markov chain is that most reads in each of the m groups are likely from the same species and Markov chains are widely used to model the microbial genome sequences [41,42]. In brief, starting from the initially assigned reads in a group, MBBC counts 6-mer frequencies in these reads to obtain the transition matrix and the stationary probability of the Markov chain. Next, MBBC scores all reads in this group using the inferred Markov model and obtains the beta percentile of the score distribution. This percentile is used as a cutoff to determine whether a read belongs to a species. The beta used by MBBC in all tested datasets is 10%. MBBC then scores each read with m trained models and finds the model with the best score for each read. If the best score is larger than the corresponding cutoff, this read is assigned to the species corresponding to the best score. Otherwise, the read is not assigned. With all reads scored and assigned, we have a new set of *m* + *1* groups of reads and infer the Markov models for the m groups again. This process of inferring the Markov models and assigning reads is iteratively implemented, with the beta decreased to beta/2 after one iteration, until the assigned reads in the m + 1 groups do not change. With the final assigned reads, MBBC estimates the genome size of each species using the total number of k-mers in each group divided by the estimated k-mer coverage.

### Comparisons with abundancebin and MetaCluster 5.0

To run MBBC, we used the following command for each dataset: java –jar –Xmx7g MBBC.jar –i reads_file -m species_number –r read_type, where m was set to be 10, and the read_type = 1 indicates single-end reads and read_type = 0 means paired-end reads. With the known species number *m* as input, we ran AbundanceBin [14] using the command “./abundancebin -input reads_file -bin_num m”. We ran MetaCluster 5.0 [15] by the command “./ MetaCluster5_1 reads_file --Species m’” for species with the genome coverage larger than 6 first and then using the command “./ MetaCluster5_2 reads_file.2 --Species m” for the species with the genome coverage smaller than 6. When the species number was assumed to be unknown, we ran AbundanceBin and MetaCluster 5.0 using the command “./abundancebin -input reads_file -RECURSIVE_CLASSIFICATION” and “./ MetaCluster5_1 reads_file” followed by “./ MetaCluster5_2 reads_file.2”, respectively.

## Additional files

Additional file 1: Table S1. The human gut dataset from 15 randomly selected samples; **Table S2.** Mapped reads of each of the three species in the human gut dataset; **Table S3.** 12 randomly selected species from three genera; **Table S4.** 12 simulated datasets.
Additional file 2: Table S1. Read binning accuracy of MBBC on each of 12 simulated datasets; **Table S2.** MBBC predicted the genome sizes, relative abundance, and the k-mer coverage in each of 12 simulated datasets; **Table S3.** Read binning accuracy of MBBC on datasets listed in Table 1.
Additional file 3: S1. MBBC, AbundanceBin and MetaCluster on additional simulated datasets with species from different phyla; **S2.** MBBC, AbundanceBin and MetaCluster on HMP real datasets; **S3.** MBBC and AbundanceBin on HMP mock datasets; **S4.** MBBC, AbundanceBin and MetaCluster on original AMD raw read dataset; **S5.** MBBC, AbundanceBin, and MetaCluster on original human gut raw read dataset.
Additional file 4: Table S1. The number of species predicted by MetaCluster and AbundanceBin on 12 simulated datasets and 2 real datasets without inputting the correct species numbers; **Table S2.** The prediction by AbundanceBin and MetaCluster with the correct species number specified.
Additional file 5:
**The total running time of each method on different datasets.**

## Abbreviations

MBBC: Metagenomic binning based on composition
OTUs: Operative taxonomical units
