# Supplementary material for: MBBC: an efficient approach for metagenomic binning based on clustering
Source: BMC Bioinformatics. 2015 Feb 5;16:36. doi: 10.1186/s12859-015-0473-8 (PMC4339733; doi:10.1186/s12859-015-0473-8)
Supplement: Additional file 1: Table S1. — The human gut dataset from 15 randomly selected samples; Table S2. Mapped reads of each of the three species in the human gut dataset; Table S3. 12 randomly selected species from three genera; Table S4. 12 simulated datasets. [file 12859_2015_473_MOESM1_ESM.docx]

# Additional file 1

Table S1: the human gut dataset for 15 randomly selected samples

Table S2: Mapped reads of each of three species for human gut dataset

Table S3: 12 randomly selected species from three genera

Table S4: 12 simulated datasets

Table S1: the human gut dataset for 15 randomly selected samples

| # | name | # reads (base pairs) |
| --- | --- | --- |
| 1 | MH0002_081203_clear | 7276422 |
| 2 | MH0002_081224.clean | 18620790 |
| 3 | MH0025_090120 | 22810856 |
| 4 | MH0026_081224.clean | 21744218 |
| 5 | MH0030_081224.clean | 20201454 |
| 6 | MH0032_081224.clean | 18060220 |
| 7 | MH0037_081226.clean | 12559802 |
| 8 | MH0046_081230 | 9229304 |
| 9 | MH0051_081226.clean | 16775182 |
| 10 | MH0054_081222.clean | 16967244 |
| 11 | MH0059_081230 | 16418700 |
| 12 | MH0066_081223.clean | 20294208 |
| 13 | MH0071_090104 | 19726578 |
| 14 | MH0078_081222.clean | 17089798 |
| 15 | MH0079_090104 | 19383978 |
|  | all reads | 257158754 |

Table S2: Mapped reads of each of three species for human gut dataset

|  | # mapped reads | reads length | k-mer coverage | relative abundance | genome length |
| --- | --- | --- | --- | --- | --- |
| Alistipes putredinis | 661370 | 75 bp | 8.28 | 14.12% | na |
| Ruminococcusbromii L2-63 | 780818 | 75 bp | 10.49 | 16.67% | 2249085 bp |
| Bacteroides uniformis | 3241910 | 75 bp | 18.49 | 69.21% | na |

Table S3: 12 randomly selected species from three genera

| species | genome length (bp) |
| --- | --- |
| Lactobacillus amylovorus GRL1118 | 1894401 |
| Lactobacillus gasseri ATCC 33323 | 1894360 |
| Lactobacillus ruminis ATCC 27782 | 2066652 |
| Lactobacillus sakei subsp. sakei 23K | 1884661 |
| Spiroplasma apis B31 | 1160554 |
| Spiroplasma diminutum CUAS-1 | 945296 |
| Spiroplasma syrphidicola EA-1 | 1107344 |
| Spiroplasma taiwanense CT-1 | 1075140 |
| Bartonella australis AustNH1 | 1596490 |
| Bartonella bacilliformis KC583 | 1445021 |
| Bartonella clarridgeiae 73 | 1522743 |
| Bartonella henselae str. Houston-1 | 1931047 |

Table S4: 12 simulated datasets

Each species in each dataset is named by the first two letters of their genus names, one letter from species name and then following by the reads coverage; the datasets were generated from reads with errors (~1%) and no errors.

| lag5lar11las24 |
| --- |
| lag4lar7las12 |
| laa4lag8lar15las30 |
| laa4lag8lar15las30_no_errors |
| spa4spd9sps18 |
| spa5spd8sps15 |
| spa4spd8sps18spt32 |
| spa4spd8sps18spt32_no_errors |
| baa3bab7bac15 |
| baa6bab10bac18 |
| baa5bab10bac18bah30 |
| baa5bab10bac18bah30_no_errors |
