# Supplementary material for: MBBC: an efficient approach for metagenomic binning based on clustering
Source: BMC Bioinformatics. 2015 Feb 5;16:36. doi: 10.1186/s12859-015-0473-8 (PMC4339733; doi:10.1186/s12859-015-0473-8)
Supplement: Additional file 2: Table S1. — Read binning accuracy of MBBC on each of 12 simulated datasets; Table S2. MBBC predicted the genome sizes, relative abundance, and the k-mer coverage in each of 12 simulated datasets; Table S3. Read binning accuracy of MBBC on datasets listed in Table 1. [file 12859_2015_473_MOESM2_ESM.docx]

**Additional file 2**

Table S1: Read binning accuracy by MBBC on each of 12 simulated datasets

Table S2: MBBC predicted the genome sizes, relative abundance, and the k-mer coverage in each of 12 simulated datasets

Table S3: The read binning accuracy of MBBC on datasets listed in Table 1

Table S1: MBBC read binning accuracy for each of 12 simulated datasets

| dataset | Binning accuracy |
| --- | --- |
| lag5lar11las24 | 91.34% |
| lag4lar7las12 | 78.97% |
| laa4lag8lar15las30 | 86.43% |
| spa4spd9sps18 | 89.58% |
| spa5spd8sps15 | 82.01% |
| spa4spd8sps18spt32 | 87.35% |
| baa3bab7bac15 | 79.55% |
| baa6bab10bac18 | 75.80% |
| baa5bab10bac18bah30 | 75.71% |
| laa4lag8lar15las30_no_errors | 87.13% |
| spa4spd8sps18spt32_no_errors | 89.09% |
| baa5bab10bac18bah30_no_errors | 79.90% |

Table S2: MBBC predicted genome size, relative abundance, and the k-mer coverage for each of 12 simulated datasets

|  | Predicted genome size | Predicted relative abundance | Predicted k-mer coverage | Real genome size | Real relative abundance | Real k-mer coverage |
| --- | --- | --- | --- | --- | --- | --- |
| lag5lar11las24 | 2554663 | 14.12% | 3.42 | 1894360 | 12.23% | 4.09 |
|  | 2195883 | 27.37% | 7.72 | 2066652 | 29.36% | 8.13 |
|  | 2147938 | 58.51% | 16.87 | 1884661 | 58.41% | 16.31 |
| lag4lar7las12 | 2734920 | 24.94% | 3.26 | 1894360 | 16.97% | 3.57 |
|  | 1526037 | 25.95% | 6.08 | 2066652 | 32.39% | 5.39 |
|  | 1979027 | 49.11% | 8.87 | 1884661 | 50.64% | 8.55 |
| spa4spd9sps18 | 1365941 | 15.16% | 2.94 | 1160554 | 14.03% | 3.49 |
|  | 994578 | 24.84% | 6.61 | 945296 | 25.72% | 6.48 |
|  | 1238383 | 60.00% | 12.82 | 1107344 | 60.25% | 12.48 |
| spa5spd8sps15 | 1607360 | 27.03% | 4.03 | 1160554 | 19.36% | 4.01 |
|  | 682864 | 20.95% | 7.36 | 945296 | 25.23% | 5.83 |
|  | 1139322 | 52.02% | 10.95 | 1107344 | 55.41% | 10.53 |
| baa3bab7bac15 | 1811825 | 16.03% | 2.67 | 1596490 | 12.69% | 3.03 |
|  | 1107173 | 19.97% | 5.45 | 1445021 | 26.80% | 5.29 |
|  | 1795434 | 63.99% | 10.76 | 1522743 | 60.51% | 10.68 |
| baa6bab10bac18 | 2075980 | 22.62% | 4.48 | 1596490 | 18.62% | 4.61 |
|  | 1102915 | 20.88% | 7.79 | 1445021 | 28.09% | 7.29 |
|  | 1779465 | 56.50% | 13.07 | 1522743 | 53.29% | 12.67 |
| laa4lag8lar15las30 | 3019238 | 11.80% | 3.45 | 1894401 | 6.87% | 3.5 |
|  | 1423375 | 11.25% | 6.97 | 1894360 | 13.74% | 5.97 |
|  | 2033991 | 25.52% | 11.07 | 2066652 | 28.11% | 10.9 |
|  | 2138138 | 51.43% | 21.22 | 1884661 | 51.27% | 19.76 |
| laa4lag8lar15las30_no errors | 2216132 | 8.89% | 3.54 | 1894401 | 6.87% | 3.8 |
|  | 1468219 | 11.73% | 7.05 | 1894360 | 13.74% | 6.77 |
|  | 1935123 | 27.05% | 12.33 | 2066652 | 28.11% | 12.76 |
|  | 1912023 | 52.33% | 24.15 | 1884661 | 51.27% | 24.67 |
| spa4spd8sps18spt32 | 1498994 | 9.42% | 3.34 | 1160554 | 6.98% | 3.49 |
|  | 825923 | 10.35% | 6.67 | 945296 | 11.36% | 5.83 |
|  | 1138156 | 27.91% | 13.05 | 1107344 | 29.95% | 12.48 |
|  | 1212248 | 52.33% | 22.98 | 1075140 | 51.70% | 20.52 |
| spa4spd8sps18spt32_no errors | 977407 | 6.30% | 3.43 | 1160554 | 6.98% | 3.78 |
|  | 877835 | 11.23% | 6.81 | 945296 | 11.36% | 6.6 |
|  | 1058603 | 28.99% | 14.58 | 1107344 | 29.95% | 14.71 |
|  | 1100332 | 53.48% | 25.87 | 1075140 | 51.70% | 26.62 |
| baa5bab10bac18bah30 | 2974101 | 14.26% | 4.13 | 1596490 | 7.41% | 4.05 |
|  | 1298657 | 13.24% | 8.79 | 1445021 | 13.41% | 7.29 |
|  | 1397781 | 23.56% | 14.53 | 1522743 | 25.43% | 12.67 |
|  | 1930614 | 48.94% | 21.86 | 1931047 | 53.75% | 20.22 |
| baa5bab10bac18bah30_no errors | 1774118 | 9.07% | 4.41 | 1596490 | 7.41% | 4.45 |
|  | 1202710 | 12.58% | 9.02 | 1445021 | 13.41% | 8.36 |
|  | 1397938 | 25.19% | 15.54 | 1522743 | 25.43% | 14.93 |
|  | 1857680 | 53.15% | 24.67 | 1931047 | 53.75% | 25.44 |

Table S3: MBBC read binning accuracy for the datasets in Table 1

| dataset | Binning accuracy |
| --- | --- |
| spa4spd8sps18spt32 | 87.35% |
| spa4spd8sps18 | 89.67% |
| spa5spd8sps15 | 82.01% |
| spa5baa8sps15 | 85.39% |
