# Supplementary material for: MBBC: an efficient approach for metagenomic binning based on clustering
Source: BMC Bioinformatics. 2015 Feb 5;16:36. doi: 10.1186/s12859-015-0473-8 (PMC4339733; doi:10.1186/s12859-015-0473-8)
Supplement: Additional file 3: S1. — MBBC, AbundanceBin and MetaCluster on additional simulated datasets with species from different phyla; S2. MBBC, AbundanceBin and MetaCluster on HMP real datasets; S3. MBBC and AbundanceBin on HMP mock datasets; S4. MBBC, AbundanceBin and MetaCluster on original AMD raw read dataset; S5. MBBC, AbundanceBin, and MetaCluster on original human gut raw read dataset. [file 12859_2015_473_MOESM3_ESM.docx]

# Additional file 3

**S1. MBBC, AbundanceBin and MetaCluster on additional simulated datasets with species from different phyla**

S1.1 Reads assignment accuracy

S1.2 The predicted genome size, relative abundance, and the k-mer coverage from MBBC and AbundanceBin

**S2. MBBC, AbundanceBin and MetaCluster on HMP real datasets**

S2.1 Reads assignment accuracy

S2.2 The predicted genome size, relative abundance, and the k-mer coverage from MBBC and AbundanceBin;

S2.3 MBBC results when using different initial species numbers

**S3. MBBC and AbundanceBin on HMP mock datasets**

S3.1 Reads assignment accuracy

S3.2 The predicted genome size, relative abundance, and the k-mer coverage from MBBC and AbundanceBin

S3.3 MBBC on original HMP mock datasets

**S4. MBBC, AbundanceBin and MetaCluster on original AMD dataset**

**S5. MBBC, AbundanceBin, and MetaCluster on original human gut dataset.**

**S1. MBBC, AbundanceBin and MetaCluster on additional simulated datasets with species from different phyla.**

Species from different phyla in the NCBI Microbial Genome Database (<http://www.ncbi.nlm.nih.gov/genomes/MICROBES/microbial_taxtree.html>) were randomly chosen to generate additional simulated datasets. Three datasets were generated, each of which contain three or four species. All species used were listed here: Aeropyrum camini SY1 JCM 12091 uid222311, Bacillus cytotoxicus NVH 391 98 uid58317, candidate division SR1 bacterium RAAC1 SR1 1 uid230714, Aquifex aeolicus VF5 uid57765 Chlamydia muridarum Nigg uid57785, Corynebacterium glutamicum R uid58897, Kosmotoga olearia TBF 19 5 1 uid59205, Aequorivita sublithincola DSM 14238 uid168181, Archaeoglobus fulgidus DSM 4304 uid57717, Calditerrivibrio nitroreducens DSM 19672 uid60821. The species Corynebacterium_glutamicum_R_uid58897 was from Phylum Actinobacteria, which contain GC biased reads. We then generated paired-end reads using MetaSim for each of the three or four species in a dataset, with the same genome coverage as used in Table 1 in the paper. We specified the read length to be 75 base pairs and simulated reads with the empirical error model in MetaSim. The results were in the following two tables. The names in the tables followed the same format we used in the paper. That is, the first two letter of the first word concatenated with the first letter of the second word in the species name was used as the species name.

S1.1 Reads assignment accuracy

| Dataset | MBBC (m=10) | MetaCluster (m is known) | AbundanceBin (m is known) |
| --- | --- | --- | --- |
| aec4bac8cad18 | 90.83% | 51.74% | 79.42% |
| chm4aqa8cog18koo32 | 94.13% | 86.52% | 72.67% |
| aes5arf8can15 | 90.75% | 46.57% | 62.38% |

S1.2 The predicted genome size, relative abundance, and the k-mer coverage from MBBC and AbundanceBin (MetaCluster does not provide the prediction of these parameters).

| MBBC results (m=10) | | | | AbundanceBin results (m is known) | | | | Real data | |
| --- | --- | --- | --- | --- | --- | --- | --- | --- | --- |
| datasets | Predicted genome size | Predicted relative abundance | Predicted k-mer coverage | Predicted genome size | Predicted relative abundance | Predicted k-mer coverage | Real genome size | Real relative abundance | Real k-mer coverage |
| aec4bac8cad18 | 2439558.54 | 12.39% | 2.45 | 14078790.17 | 5.77% | 1.16 | 1595994 | 10.59% | 3.44 |
|  | 4093351.18 | 47.12% | 5.55 | 4747113.02 | 66.85% | 9.6 | 4087024 | 54.24% | 5.91 |
|  | 1556990.19 | 40.49% | 12.54 | 1191417.31 | 27.39% | 23.65 | 1177760 | 35.17% | 12.39 |
| chm4aqa8cog18koo32 | 2114455.31 | 5.18% | 2.94 | 33085519.71 | 2.16% | 1.1 | 1072931 | 2.86% | 3.45 |
|  | 1694191.59 | 8.07% | 5.72 | 1951520.62 | 16.39% | 9.63 | 1551335 | 8.27% | 5.77 |
|  | 3671354.93 | 38.63% | 12.63 | 3475605.23 | 48.75% | 23.46 | 3314174 | 39.76% | 12.44 |
|  | 2561756.15 | 48.12% | 22.55 | 1983461.47 | 32.69% | 44 | 2302126 | 49.10% | 21.16 |
| aes5arf8can15 | 3880941.6 | 27.77% | 3.86 | 15659129.27 | 4.93% | 1.15 | 3520671 | 26.12% | 4.00 |
|  | 1986885.09 | 25.53% | 6.93 | 4767405.54 | 57.10% | 8.42 | 2178400 | 25.86% | 5.80 |
|  | 2316543.24 | 46.70% | 10.87 | 2178181.19 | 37.96% | 19.53 | 2157835 | 48.02% | 10.58 |

**S2: MBBC, AbundanceBin and MetaCluster on HMP real datasets.**

We randomly selected three datasets from the Human Microbiome Project (http://hmpdacc.org/HMSCP/): SRS013542, SRS017080 and SRS013705. These datasets contained mapped paired-end reads. For each dataset, we used the mapped reads information they provided to calculate the k-mer coverage for each species. We then selected three or four species that have highest k-mer coverage and highest relative abundance in each dataset. Each of the three generated datasets was composed of reads from these selected species. Because MetaCluster required that all reads have equal length, we cut the reads to be 75 base pairs.

S2.1 Reads assignment accuracy

| datasets | MBBC | MetaCluster (m is known) | AbundanceBin (m is known) |
| --- | --- | --- | --- |
| SRS013542 | 83.60% | 42.34% | 65.98% |
| SRS017080 | 72.68% | 65.78% | 65.60% |
| SRS013705 | 78.16% | 67.01% | 44.18% |

S2.2 The predicted genome size, relative abundance, and the k-mer coverage from MBBC and AbundanceBin

| MBBC results | | | | AbundanceBin results (m is known) | | | Real data | | |
| --- | --- | --- | --- | --- | --- | --- | --- | --- | --- |
| datasets | Predicted genome size | Predicted relative abundance | Predicted k-mer coverage | Predicted genome size | Predicted relative abundance | Predicted k-mer coverage | Species | Real relative abundance | Real k-mer coverage |
| SRS013542 | 3013014.58 | 15.89% | 3.16 | 7278053.67 | 10.71% | 2.08 | Prevotella_timonensis | 8.32% | 4.62 |
|  | 1055820.12 | 13.38% | 7.6 | 2859624.66 | 38.66% | 14.58 | Atopobium_vaginae | 11.81% | 9.07 |
|  | 708216.32 | 15.32% | 12.97 | 966392.16 | 50.63% | 56.92 | Gardnerella_vaginalis | 79.87% | 28.18 |
|  | 1390796.12 | 55.41% | 23.9 |  |  |  |  |  |  |
| SRS017080 | 4611172.74 | 28.66% | 3.25 | 10232486.5 | 16.37% | 1.91 | Veillonella_dispar | 10.04% | 4.04 |
|  | 628620.41 | 11.82% | 9.83 | 1851573.49 | 36.49% | 17.3 | Streptococcus_pneumoniae | 20.56% | 9.91 |
|  | 1780668.57 | 59.52% | 17.49 | 1100613.52 | 47.13% | 41.75 | Rothia_mucilaginosa | 69.40% | 23.81 |
| SRS013705 | 3142540.31 | 9.55% | 4.82 | 369661.72 | 3.58% | 2.5 | Prevotella_tannerae | 1.51% | 4.07 |
|  | 875747.63 | 6.67% | 12.08 | 2088088.63 | 23.27% | 18.2 | Leptotrichia_buccalis | 2.86% | 10.15 |
|  | 2175197.92 | 32.54% | 23.72 | 4068813.93 | 25.65% | 61.41 | Campylobacter_concisus | 38.61% | 23.75 |
|  | 2083794.8 | 51.24% | 38.99 | 10384164.72 | 47.50% | 183.15 | Fusobacterium_sp | 57.02% | 44.39 |

MBBC predicted four species for the dataset SRS013542, but by looking at the number of real reads in each predicted species as shown in the following table, we found that the last two predicted species mainly contain reads from the third species.

| # real reads in each predicted species | | | | |
| --- | --- | --- | --- | --- |
| Species | Predicted species1 | Predicted species2 | Predicted species3 | Predicted species4 |
| Prevotella_timonensis | 150776 | 5942 | 5022 | 4814 |
| Atopobium_vaginae | 76330 | 138290 | 11036 | 10626 |
| Gardnerella_vaginalis | 90932 | 123488 | 290594 | 1093298 |

S2.3 MBBC results when using different initial species numbers

We did testings for SRS013542 by setting the initial species number to be 9, 10, 11, 12, 13 and 14. Different initial species numbers will result in inexact results, but MBBC can generally divide the reads into same number of groups. MBBC results were shown in the following table:

| initial species number | Predicted # species | Predicted genome size | Predicted relative abundance | Predicted k-mer coverage |
| --- | --- | --- | --- | --- |
| m=9 | 1 | 3191635.65 | 18.94% | 3.56 |
|  | 2 | 1147967.42 | 16.24% | 8.49 |
|  | 3 | 486258.74 | 12.48% | 15.38 |
|  | 4 | 1177873.97 | 52.34% | 26.66 |
| m=10 | 1 | 3013014.58 | 15.89% | 3.16 |
|  | 2 | 1055820.12 | 13.38% | 7.6 |
|  | 3 | 708216.32 | 15.32% | 12.97 |
|  | 4 | 1390796.12 | 55.41% | 23.9 |
| m=11 | 1 | 2986751.53 | 15.07% | 3.03 |
|  | 2 | 1066440.45 | 12.97% | 7.3 |
|  | 3 | 934883.17 | 19.32% | 12.39 |
|  | 4 | 959556.52 | 52.63% | 32.91 |
| m=12 | 1 | 2947407.59 | 14.73% | 3.00 |
|  | 2 | 1078248.03 | 13.00% | 7.23 |
|  | 3 | 943216.24 | 19.31% | 12.28 |
|  | 4 | 972443.83 | 52.96% | 32.67 |
| m=13 | 1 | 3023343.09 | 14.63% | 2.90 |
|  | 2 | 1043023.55 | 12.22% | 7.03 |
|  | 3 | 996299.67 | 19.86% | 11.95 |
|  | 4 | 1000100.29 | 53.29% | 31.97 |
| m=14 | 1 | 3028093.36 | 14.63% | 2.90 |
|  | 2 | 1044348.96 | 12.22% | 7.02 |
|  | 3 | 997265.96 | 19.85% | 11.94 |
|  | 4 | 1001476.56 | 53.30% | 31.93 |

**S3. MBBC and AbundanceBin on HMP mock datasets;**

We downloaded reads from HMP even and staggered community (<http://www.hmpdacc.org/HMMC/>): SRR172902 and SRR172902. These datasets contain single-end reads. All reads were mapped to 22 reference genomes using software SOAP with default parameters. For each dataset, we selected three species that have highest relative abundance in each dataset. Each of the two generated datasets was composed of reads from three species. Because MetaCluster can only be applied to paired-end reads, we did comparisons between MBBC and AbundanceBin.

S3.1 Reads assignment accuracy

|  | MBBC results | AbundanceBin results (m is known) |
| --- | --- | --- |
| SRR172902 | 84.98% | 70.55% |
| SRR172903 | 97.34 | 80.24% |

S3.2 The predicted genome size, relative abundance, and the k-mer coverage from MBBC and AbundanceBin

| MBBC results | | | | AbundanceBin (m is known) | | | Real data | | |
| --- | --- | --- | --- | --- | --- | --- | --- | --- | --- |
| datasets | Predicted genome size | Predicted relative abundance | Predicted k-mer coverage | Predicted genome size | Predicted relative abundance | Predicted k-mer coverage | Species | Real relative abundance | Real k-mer coverage |
| SRR172902 | 6522002.11 | 22.71% | 5.75 | 12571565.85 | 5.62% | 1.56 | Bacteroides_vulgatus | 17.17% | 5.72 |
|  | 3133606.91 | 20.48% | 10.80 | 7114833.49 | 47.03% | 8.78 | Acinetobacter_baumannii | 22.30% | 9.03 |
|  | 1642666.08 | 22.55% | 22.68 | 2089533.66 | 47.35% | 34.54 | Deinococcus_radiodurans | 60.53% | 25.88 |
|  | 1593666.19 | 34.27% | 35.52 |  |  |  |  |  |  |
| SRR172903 | 3929542.11 | 14.14% | 5.92 | 13169922.02 | 6.32% | 1.44 | Rhodobacter_sphaeroides | 28.25% | 9.58 |
|  | 2231651.71 | 16.12% | 11.89 | 7728299.21 | 84.90% | 15.80 | Staphylococcus_aureus | 33.51% | 20.33 |
|  | 5822095.65 | 69.74% | 19.71 | 35320.78 | 8.78% | 356.21 | Staphylococcus_epidermidis | 38.24% | 22.58 |

For SRR172902, MBBC predicted four species, but by looking at the number of real reads in each predicted species, we found that the last two predicted species mainly contain reads from the third species. For SRR172903, two of the three species have very close k-mer coverage, these two groups of reads were clustered together to calculate the accuracy for both software.

S3.3 MBBC on original HMP mock datasets

To show the performance of MBBC on the original data, we ran MBBC on the original HMP mock datasets. The predicted relative abundance and k-mer coverage of MBBC were shown below:

| SRR172902 | | | | | |
| --- | --- | --- | --- | --- | --- |
| Predicted genome size | 17563305.42 | 10981106.98 | 1335932.21 | 1542912.02 | 1117717.77 |
| Predicted relative abundance | 22.99% | 32.33% | 8.88% | 16.97% | 18.83% |
| Predicted k-mer coverage | 3.67 | 8.26 | 18.63 | 30.83 | 47.23 |
| SRR172903 | | | | | |
| Predicted genome size | 8270207.75 | 4846536.17 | 3239045.8 | 3055915.66 |  |
| Predicted relative abundance | 16.16% | 21.20% | 26.17% | 36.47% |  |
| Predicted k-mer covearge | 4.51 | 10.09 | 18.63 | 27.53 |  |

The real data for the mapped HMP mock datasets were shown in the following table. The species in each dataset can be grouped according to their similar k-mer coverage. We shaded the predicted species and real data that have similar k-mer coverage with the same color. MBBC can generally divide the reads into groups that have similar k-mer coverage.

| SRR172902 | | | SRR172903 | | |
| --- | --- | --- | --- | --- | --- |
| Species | Real k-mer coverage | Real relative abundance | Species | Real k-mer coverage | Real relative abundance |
| Deinococcus_radiodurans | 25.88 | 35.67% | Staphylococcus_epidermidis | 22.58 | 27.28% |
| Acinetobacter_baumannii | 9.03 | 13.14% | Staphylococcus_aureus | 20.33 | 23.91% |
| Staphylococcus_epidermidis | 6.48 | 5.64% | Streptococcus_mutans | 10.32 | 9.45% |
| Bacteroides_vulgatus | 5.72 | 10.12% | Neisseria_meningitidis | 9.80 | 0.20% |
| Streptococcus_pneumoniae | 5.39 | 3.89% | Rhodobacter_sphaeroides | 9.58 | 20.16% |
| Helicobacter_pylori | 5.29 | 3.00% | Enterococcus_faecalis | 8.58 | 0.01% |
| Propionibacterium_acnes | 4.64 | 4.06% | Methanobrevibacter_smithii | 6.11 | 4.91% |
| Neisseria_meningitidis | 4.18 | 2.53% | Lactobacillus_gasseri | 5.31 | 0.01% |
| Staphylococcus_aureus | 3.92 | 3.02% | Streptococcus_pneumoniae | 4.67 | 0.01% |
| Actinomyces_odontolyticus | 3.50 | 2.26% | Escherichia_coli | 4.39 | 8.31% |
| Streptococcus_mutans | 3.39 | 1.92% | Listeria_monocytogenes | 3.28 | 0.09% |
| Rhodobacter_sphaeroides | 3.08 | 2.95% | Bacillus_cereus | 2.43 | 0.36% |
| Clostridium_beijerinckii | 3.06 | 4.25% | Pseudomonas_aeruginosa | 2.42 | 2.03% |
| Listeria_monocytogenes | 2.95 | 2.08% | Clostridium_beijerinckii | 2.33 | 1.59% |
| Streptococcus_agalactiae | 2.72 | 0.10% | Streptococcus_agalactiae | 2.33 | 0.58% |
| Enterococcus_faecalis | 2.65 | 1.40% | Deinococcus_radiodurans | 2.20 | 0.23% |
| Methanobrevibacter_smithii | 2.55 | 0.76% | Acinetobacter_baumannii | 2.18 | 0.51% |
| Lactobacillus_gasseri | 2.50 | 0.03% | Helicobacter_pylori | 2.08 | 0.11% |
| Escherichia_coli | 2.47 | 1.63% | Propionibacterium_acnes | 2.07 | 0.22% |
| Bacillus_cereus | 2.24 | 0.88% | Bacteroides_vulgatus | 2.03 | 0.03% |
| Pseudomonas_aeruginosa | 2.20 | 0.67% | Actinomyces_odontolyticus | 2.00 | 0.01% |
| Candida albicans | 2.00 | 0.00% | Candida albicans | 2.00 | 0.00% |

**S4. MBBC, AbundanceBin on original AMD dataset;**

As described in the paper, we have 166,715 reads left after quality filtering for the Acid Mine Drainage (AMD) dataset downloaded from <http://www.ncbi.nlm.nih.gov/books/NBK6860/>. We did the comparisons using these 166,715 reads.

To compare with our prediction, we need to know the real species present and their abundance. To get the real data for this dataset, all reads were mapped to the five main species known in literature using the MuMmer software with the default parameters. About half of the reads were mapped to the five species. The mapped reads from these five species can be further clustered into three main groups based on their k-mer coverage: the first two groups contain reads from Leptospirillum_sp_Group_II and Ferroplasma_sp_Type_II, respectively, and the third group contain reads from the remaining three species.

Using the original 166,715 reads, MBBC successfully predicted three species, while AbundanceBin could not have results when setting input number of species to be 3 or 5.

|  | Predicted genome size | Predicted relative abundance | Predicted k-mer coverage | Accuracy |
| --- | --- | --- | --- | --- |
| MBBC results | 5455354.93 | 22.01% | 4.39 | 67.60% |
|  | 4103623.62 | 44.67% | 11.59 |  |
|  | 1710919.04 | 33.32% | 20.93 |  |
| AbundanceBin results (m=3 or 5) | nan | | | |
| Real data | Species | Real relative abundance | Real k-mer coverage | |
|  | Leptospirillum_sp_Group_II | 40.45% | 7.35 | |
|  | Ferroplasma_sp_Type_II | 16.63% | 5.14 | |
|  | Thermoplasmatales_archaeon_Gpl_G-plasma | 18.57% | 3.61 | |
|  | Ferroplasma_acidarmanus_Type_I | 10.58% | 3.16 | |
|  | Leptospirillum_sp_Group_III | 13.77% | 3.09 | |

**S5. MBBC, AbundanceBin, and MetaCluster on original human gut dataset.**

We randomly selected one human gut sample from <ftp://public.genomics.org.cn/BGI/gutmeta/High_quality_reads/>, which has paired-end reads of 75 base pairs long. The original 24,737,448 reads from sample MH0047 were used to do comparisons. To compare with our prediction, we need to know the real species present and their abundance. Because we don’t know how many species were in the sample, we only consider the top 10 frequent species among individuals of cohort which is described in the paper Qin, Junjie, et al. "A human gut microbial gene catalogue established by metagenomic sequencing." Nature 464.7285 (2010): 59-65. To get actual information about the present species in this dataset, all reads were mapped to the 10 species using Blat with identity=95%. Only about 5% of reads were mapped to these 10 species.

MBBC can predict five species from the original 24,737,448 reads in the sample MH0047.

| MBBC results | | | | | |
| --- | --- | --- | --- | --- | --- |
| Predicted genome size | 95533894.19 | 12921769.13 | 6943407.73 | 4544952.25 | 2318859.39 |
| Predicted relative abundance | 37.59% | 15.13% | 15.89% | 17.38% | 14.01% |
| Predicted k-mer coverage | 2.92 | 8.69 | 16.98 | 28.38 | 44.82 |

The species can be grouped according to their similar k-mer coverage. We shaded the predicted species and real data that have similar k-mer coverage with the same color. By combining the group of reads that have similar k-mer coverage, MBBC can achieve accuracy by 73.51%. The number of real reads in each predicted species was shown below:

| # real reads in each predicted species | | | | | | |
| --- | --- | --- | --- | --- | --- | --- |
| Species | Predicted species1 | Predicted species2 | Predicted species3 | Predicted species4 | Predicted species5 | Real k-mer coverage |
| Parabacteroides_merdae_ATCC_43184 | 57195 | 11582 | **9730** | **17260** | **75297** | 10.86 |
| Bacteroides_thetaiotaomicron_VPI-5482 | 28628 | 9029 | **8777** | **7267** | **14985** | 10.43 |
| Dorea_longicatena_DSM_13814 | **10456** | **10313** | 4349 | 2948 | 3889 | 8.89 |
| Ruminococcus_torques_L2-14 | **24858** | **20754** | 6713 | 4293 | 6883 | 7.98 |
| Alistipes_putredinis_DSM_17216 | **177951** | **83797** | 18129 | 18369 | 15958 | 7.55 |
| Clostridium_sp._SS2.1 | **11399** | **10656** | 2010 | 771 | 3431 | 6.67 |
| Eubacterium_hallii_DSM_3353 | **16893** | **11767** | 2434 | 1020 | 4311 | 5.80 |
| Bacteroides_uniformis_ATCC_8492 | **83547** | **10234** | 13112 | 11021 | 21060 | 4.75 |
| Ruminococcus_bromii_L2-63 | **83847** | **12414** | 3074 | 2188 | 15093 | 4.42 |
| Bacteroides_caccae_ATCC_43185 | **98235** | **11506** | 7228 | 3529 | 14302 | 3.34 |

For MetaCluster, we set the input number of species to be 10. Only about 28% of reads can be assigned by MetaCluster. The accuracy is about 10% if we consider there are two main groups of reads. AbundanceBin has difficulty in converging or provides no result when the input number of species is 10 or 2.
