# Supplementary material for: MBBC: an efficient approach for metagenomic binning based on clustering
Source: BMC Bioinformatics. 2015 Feb 5;16:36. doi: 10.1186/s12859-015-0473-8 (PMC4339733; doi:10.1186/s12859-015-0473-8)
Supplement: Additional file 4: Table S1. — The number of species predicted by MetaCluster and AbundanceBin on 12 simulated datasets and 2 real datasets without inputting the correct species numbers; Table S2. The prediction by AbundanceBin and MetaCluster with the correct species number specified. [file 12859_2015_473_MOESM4_ESM.docx]

**Additional file 3**

Table S1: The number of species predicted by MetaCluster and AbundanceBin on 12 simulated datasets and 2 simplified real datasets without inputting the correct species numbers.

Table S2: The prediction by AbundanceBin and MetaCluster with the correct species number specified.

Table S1: The number of species predicted by MetaCluster and AbundanceBin on 12 simulated datasets and 2 simplified real datasets without inputting the correct species numbers.

| dataset | MetaCluster | AbundanceBin |
| --- | --- | --- |
| spa4spd9sps18 | 68 clusters | Nan |
| lag5lar11las24 | 7 clusters | 0 bins |
| spa5spd8sps15 | 13 clusters | Nan |
| lag4lar7las12 | 86 clusters | Nan |
| baa6bab10bac18 | 9 clusters | 2 bins |
| spa4spd8sps18spt32 | 6 clusters | 0 bins |
| laa4lag8lar15las30 | 10 clusters | 0 bins |
| baa5bab10bac18bah30 | 5 clusters | 0 bins |
| laa4lag8lar15las30_no errors | 8 clusters | no results |
| baa5bab10bac18bah30_no errors | 9 clusters | no results |
| spa4spd8sps18spt32_no errors | 149 clusters | 4 species |
| baa3bab7bac15 | 11 clusters | 4 species |
| AMD | NA | 2 species |
| human gut dataset | 572 clusters | Nan |

Table S2: The prediction by AbundanceBin and MetaCluster with the correct species number specified.

|  | AbundanceBin performance | | | MetaCluster performance | Real data | | |
| --- | --- | --- | --- | --- | --- | --- | --- |
|  | Predicted genome size | Predicted relative abundance | Predicted k-mer coverage | Predicted relative abundance | Real genome size | Real relative abundance | Real k-mer coverage |
| spa4spd9sps18 | 7750950.95 | 6.05% | 1.18 | 11.28% | 1160554 | 14.03% | 3.49 |
|  | 1418542.68 | 40.29% | 8.98 | 38.45% | 945296 | 25.72% | 6.48 |
|  | 1224947.37 | 53.65% | 22.44 | 50.27% | 1107344 | 60.25% | 12.48 |
| spa5spd8sps15 | 6845819.49 | 4.07% | 1.13 | 8.66% | 1160554 | 19.36% | 4.01 |
|  | 1745169.08 | 50.79% | 8.62 | 45.09% | 945296 | 25.23% | 5.83 |
|  | 1146773.43 | 45.14% | 19.19 | 46.26% | 1107344 | 55.41% | 10.53 |
| lag5lar11las24 | 17586263.31 | 4.16% | 1.14 | 8.05% | 1894360 | 12.23% | 4.09 |
|  | 3023119.28 | 44.84% | 11.36 | 26.86% | 2066652 | 29.36% | 8.13 |
|  | 1958769.41 | 51.00% | 31.26 | 65.09% | 1884661 | 58.41% | 16.31 |
| lag4lar7las12 | 10760829.48 | 7.37% | 1.2 | 10.48% | 1894360 | 16.97% | 3.57 |
|  | 3600901.36 | 67.00% | 9.03 | 28.24% | 2066652 | 32.39% | 5.39 |
|  | 1151774.16 | 25.64% | 18.45 | 61.28% | 1884661 | 50.64% | 8.55 |
| baa3bab7bac15 | 9359156.82 | 8.74% | 1.25 | 14.38% | 1596490 | 12.69% | 3.03 |
|  | 1821739.37 | 41.32% | 8.36 | 22.49% | 1445021 | 26.80% | 5.29 |
|  | 1453905.31 | 49.95% | 20.25 | 63.13% | 1522743 | 60.51% | 10.68 |
| baa6bab10bac18 | 11518205.6 | 2.77% | 1.09 | 11.39% | 1596490 | 18.62% | 4.61 |
|  | 2529029.77 | 51.98% | 10.4 | 17.08% | 1445021 | 28.09% | 7.29 |
|  | 1578383.63 | 45.25% | 24.03 | 63.67% | 1522743 | 53.29% | 12.67 |
| spa4spd8sps18spt32 | 14797477.77 | 3.38% | 1.14 | 8.94% | 1160554 | 6.98% | 3.49 |
|  | 1419174.26 | 19.52% | 8.72 | 8.98% | 945296 | 11.36% | 5.83 |
|  | 1154257.78 | 40.02% | 22.69 | 23.98% | 1107344 | 29.95% | 12.48 |
|  | 1058938.69 | 37.08% | 41.52 | 58.11% | 1075140 | 51.70% | 20.52 |
| spa4spd8sps18spt32_no errors | 1140816.36 | 6.22% | 6.03 | 5.55% | 1160554 | 6.98% | 3.78 |
|  | 905683.2 | 10.86% | 12.03 | 9.08% | 945296 | 11.36% | 6.6 |
|  | 1110573.48 | 30.40% | 27.08 | 25.73% | 1107344 | 29.95% | 14.71 |
|  | 1055891.46 | 52.51% | 48.79 | 59.64% | 1075140 | 51.70% | 26.62 |
| laa4lag8lar15las30 | 24855140.67 | 3.60% | 1.14 | 8.60% | 1894401 | 6.87% | 3.5 |
|  | 2558672.45 | 22.14% | 8.86 | 22.44% | 1894360 | 13.74% | 5.97 |
|  | 2140583.58 | 35.69% | 19.32 | 25.44% | 2066652 | 28.11% | 10.9 |
|  | 1790475.31 | 38.57% | 40.41 | 43.53% | 1884661 | 51.27% | 19.76 |
| laa4lag8lar15las30_no errors | 1909113.58 | 6.35% | 6.09 | 4.67% | 1894401 | 6.87% | 3.8 |
|  | 1805925.84 | 13.42% | 12.51 | 23.36% | 1894360 | 13.74% | 6.77 |
|  | 1931290.74 | 26.74% | 23.27 | 26.36% | 2066652 | 28.11% | 12.76 |
|  | 1796735.85 | 53.49% | 47.6 | 45.61% | 1884661 | 51.27% | 24.67 |
| baa5bab10bac18bah30 | 23657883.81 | 2.36% | 1.11 | 7.77% | 1596490 | 7.41% | 4.05 |
|  | 2281316.18 | 24.18% | 10.53 | 8.95% | 1445021 | 13.41% | 7.29 |
|  | 2018641.41 | 42.89% | 25.91 | 23.54% | 1522743 | 25.43% | 12.67 |
|  | 1296013.08 | 30.57% | 44.97 | 59.74% | 1931047 | 53.75% | 20.22 |
| baa5bab10bac18bah30_no errors | 2218778.19 | 11.43% | 9.14 | 4.90% | 1596490 | 7.41% | 4.45 |
|  | 1881810.62 | 28.32% | 23.53 | 8.79% | 1445021 | 13.41% | 8.36 |
|  | 1961148.7 | 55.18% | 44.62 | 30.66% | 1522743 | 25.43% | 14.93 |
|  | 75649.49 | 5.07% | 117.3 | 55.64% | 1931047 | 53.75% | 25.44 |
| AMD | 1646983.67 | 11.97% | 2.83 | NA | 1.82Mb | 29.03% | 5.14 |
|  | 2734429.83 | 88.03% | 8.5 |  | 2.23Mb | 70.97% | 7.35 |
| real human gut | 16362445.55 | 2.88% | 1.53 | 7.10% | NA | 14.12% | 8.28 |
|  | 5187924.99 | 40.34% | 17.39 | 17.66% | 2249085 | 16.67% | 10.49 |
|  | 2545199.95 | 56.78% | 57.77 | 67.54% | NA | 69.21% | 18.49 |
