# Supplementary figures and images for: MBBC: an efficient approach for metagenomic binning based on clustering
Source: BMC Bioinformatics. 2015 Feb 5;16:36. doi: 10.1186/s12859-015-0473-8 (PMC4339733; doi:10.1186/s12859-015-0473-8)

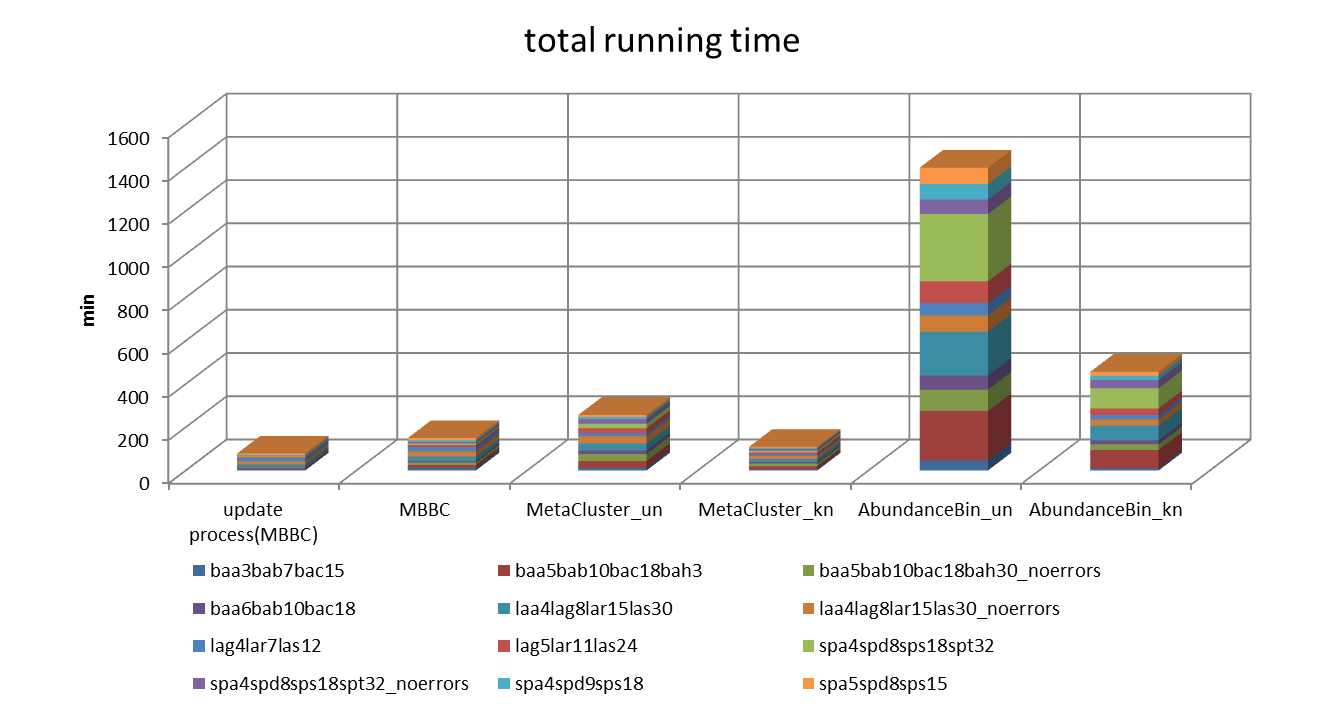

Supplement: Additional file 5: — The total running time of each method on different datasets. [file 12859_2015_473_MOESM5_ESM.tiff]
